# Novel Reassortant Highly Pathogenic H5N2 Avian Influenza Viruses in Poultry in China

**DOI:** 10.1371/journal.pone.0046183

**Published:** 2012-09-25

**Authors:** Guo Zhao, Xiaobing Gu, Xinlun Lu, Jinjin Pan, Zhiqiang Duan, Kunkun Zhao, Min Gu, Qingtao Liu, Liang He, Jian Chen, Shengqiang Ge, Yanhong Wang, Sujuan Chen, Xiaoquan Wang, Daxin Peng, Hongquan Wan, Xiufan Liu

**Affiliations:** 1 College of Veterinary Medicine, Yangzhou University, Yangzhou, Jiangsu, China; 2 Ministry of Education Key Lab for Avian Preventive Medicine, Yangzhou University, Yangzhou, Jiangsu, China; Centers for Disease Control and Prevention, United States of America

## Abstract

There has been multiple evidence that domestic poultry may act as a vessel for the generation of novel influenza A viruses. In this study, we have analyzed the evolution and pathogenicity of 4 H5N2 avian influenza viruses isolated from apparently healthy poultry from H5N1 virus endemic areas in China. Phylogenetic analysis revealed that two of these viruses, A/duck/Eastern China/1111/2011 (DK/EC/1111/11) and A/goose/Eastern China/1112/2011 (GS/EC/1112/11) were derived from reassortment events in which clade 2.3.4 highly pathogenic avian influenza (HPAI) H5N1 viruses acquired novel neuraminidase and nonstructural protein genes. Another two isolates, A/chicken/Hebei/1102/2010 (CK/HB/1102/10) and A/duck/Hebei/0908/2009 (DK/HB/0908/09), possess hemagglutinin (HA) gene belong to clade 7 H5 viruses and other genes from endemic H9N2 viruses, or from viruses of various subtypes of the natural gene pool. All of these H5N2 isolates bear characteristic sequences of HPAI virus at the cleavage site of HA, and animal experiments indicated that all of these viruses but DK/HB/0908/09 is highly pathogenic to chickens. In particular, DK/EC/1111/11 and GS/EC/1112/11 are also highly pathogenic to ducks and moderately pathogenic to mice. All of these 4 viruses were able to replicate in domestic ducks and mice without prior adaptation. The emergence of these novel H5N2 viruses adds more evidence for the active evolution of H5 viruses in Asia. The maintenance of the highly pathogenic phenotype of some of these viruses even after reassortment with a new NA subtypes, their ability to replicate and transmit in domestic poultry, and the pathogenicity in the mammalian mouse model, highlight the potential threat posed by these viruses to both veterinary and public health.

## Introduction

The Asian highly pathogenic avian influenza (HPAI) H5N1 viruses that emerged over a decade ago in China have evolved into over ten distinct phylogenetic clades on the basis of the hemagglutinin (HA) gene [1]. From 2000 onwards, various reassortant viruses were detected in geese, ducks, chickens, and other terrestrial poultry, with the HA gene derived from the A/goose/Guangdong/1/96-like (Gs/GD-like) lineage and the internal genes from other subtypes of influenza viruses [2], [3]. Recently, HPAI H5N5 virus has been identified in domestic ducks, highlighting the role of ducks in the generation of novel H5 HPAI virus with new neuraminidase (NA) subtype [4]. Among those recognized reassortants, only a few genotypes have been persistent or prevalent [5], [6]. Over the same period, HPAI H5N1 virus expanded its host range to include a variety of birds and mammalian species [6]. The endemicity in poultry and the introduction into humans of H5N1 viruses emphasize the importance of continued surveillance, isolation, and characterization of influenza virus subtypes and variants in poultry [3], [4], [5], [6].

Influenza virus of H5N2 subtype is present in wild birds usually with low pathogenicity. After introduction into domestic poultry, however, this virus may mutate into a HPAI strain [7], [8]. The acquisition of the highly pathogenic phenotype is associated with the loss of a glycosylation site at amino acid position 11, or the insertion of multiple basic residues upstream from the cleavage site, in the HA molecule [8]. In Asia, H5N2 virus was previously recorded mainly in migratory birds. In recent years, it has been isolated in poultry and mammals, including humans, pigs and dogs [9], [10], [11], [12]. Phylogenetic analyses revealed that some gene segments of the H5N2 viruses isolated in Asia were derived from the American lineage, indicating an intercontinental exchange and dissemination of these viruses [9]. Notably, all H5N2 isolates reported in Asian countries so far were of low pathogenicity.

**Figure 1:**
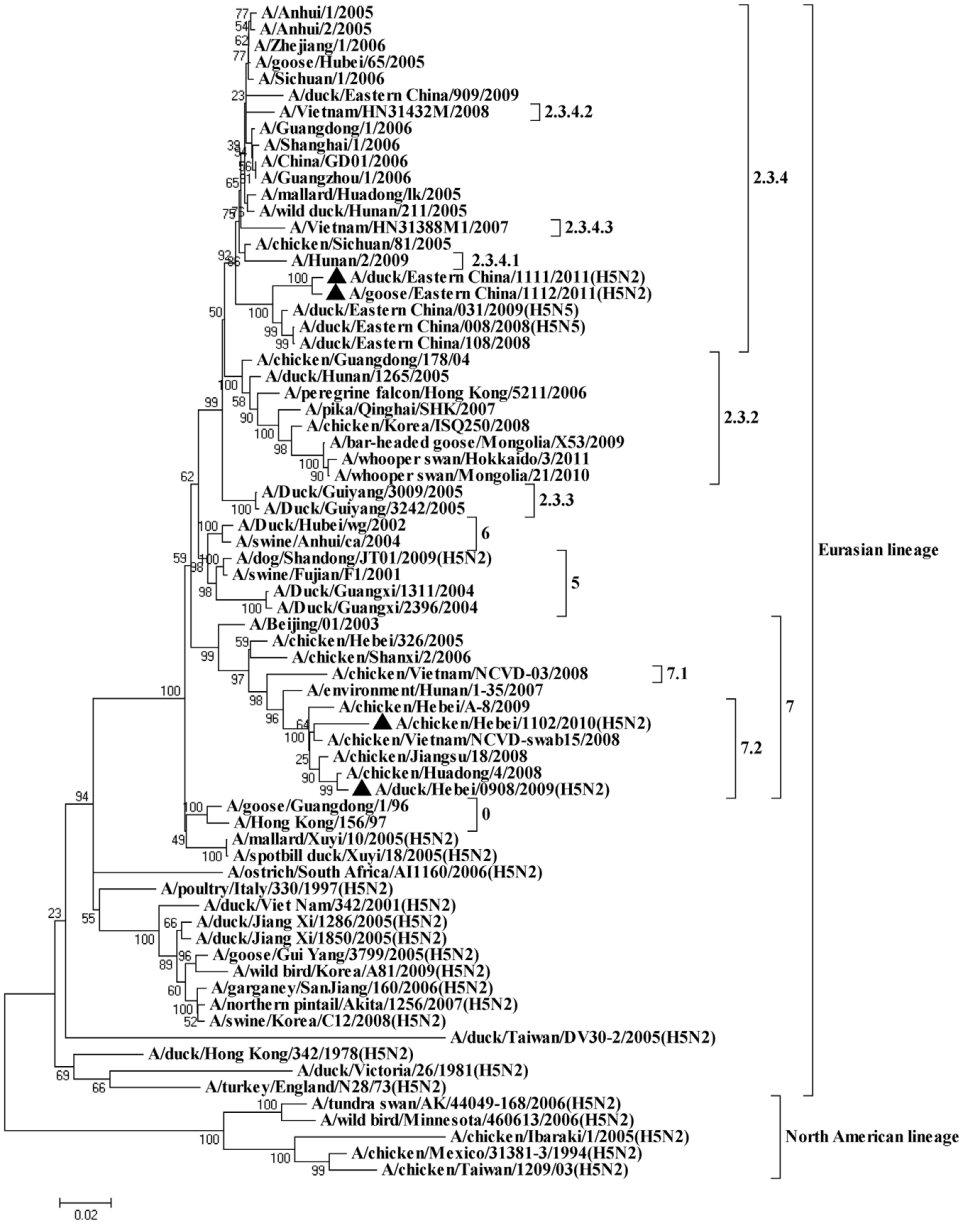
Phylogenetic trees based on the open reading frame sequences of haemagglutinin genes of H5N2 viruses in this study and those of reference strains from GenBank. Viruses highlighted with a closed triangle were those characterised in this study. The trees were constructed using the neighbour-joining method of MEGA 4.0 with 1,000 bootstrap trials performed to assign confidence to the grouping.

In this study, we characterized four novel reassortant H5N2 avian influenza viruses isolated from poultry in China, which possess HA genes closely related to the circulating HPAI H5N1 viruses. None of these isolates grouped in the HA tree (Fig. 1) with those low pathogenic H5N2 viruses previously reported in Asia [9], [10], [11], [12]. However, the pathogenicity of these viruses for chickens, domestic ducks and mice varies. Our findings also add more evidence for the active evolution of H5N1 viruses in Asia.

## Results

### Isolation and Identification of H5N2 Virus

During our routine surveillance of avian influenza viruses at a live bird market (LBM) in Eastern China, 5 H5N1, 2 H5N5 and 4 H5N2 viruses were isolated from apparently healthy domestic poultry, which have been vaccinated with an inactivated, bivalent influenza vaccine (H5N1 and H9N2). The 4 H5N2 isolates, as listed in Table 1, were abbreviated as DK/HB/0908/09, CK/HB/1102/10, DK/EC/1111/11 and GS/EC/1112/11, respectively. DK/HB/0908/09 was isolated in ducks delivered to the LBM from Hebei province, China in August 2009, while CK/HB/1102/10 was isolated from chickens from Hebei in October 2010. DK/EC/1111/11 and GS/EC/1112/11 were isolated from ducks and geese delivered from Shandong and Jiangsu province, China, respectively, in January 2011. Viruses were purified with plaque assay and used in the subsequent analysis and experiment.

**Table 1:** Characteristics of the four H5N2 influenza viruses in this study*.

Virus	Abbreviation	Connecting-peptide(HA)	EID_50_ (lg/mL)	MDT (h)	IVPI (chicken)	IVPI (duck)	MLD_50_
A/duck/Hebei/0908/2009(H5N2)	DK/HB/0908/09	PQIEGRRRKR/G	7.32	91.2	0	0	>10^7.0^
A/chicken/Hebei/1102/2010(H5N2)	CK/HB/1102/10	PQIEGRRRKR/G	7.43	64.8	2.12	0.26	>10^7.0^
A/duck/Eastern China/1111/2011(H5N2)	DK/EC/1111/11	PLREKRR-KR/G	7.32	36	2.72	2.82	10^5.5^
A/goose/Eastern China/1112/2011(H5N2)	GS/EC/1112/11	PLRGKRR-KR/G	8.50	42	2.74	2.40	10^5.2^

* EID_50_, 50% egg infectious dose; MDT, mean death time; IVPI, intravenous pathogenicity index; MLD50, 50% mouse lethal dose, expressing as the number of EID_50_ corresponding to 1 LD_50_.

### Phylogenetic Analysis

**Figure 2:**
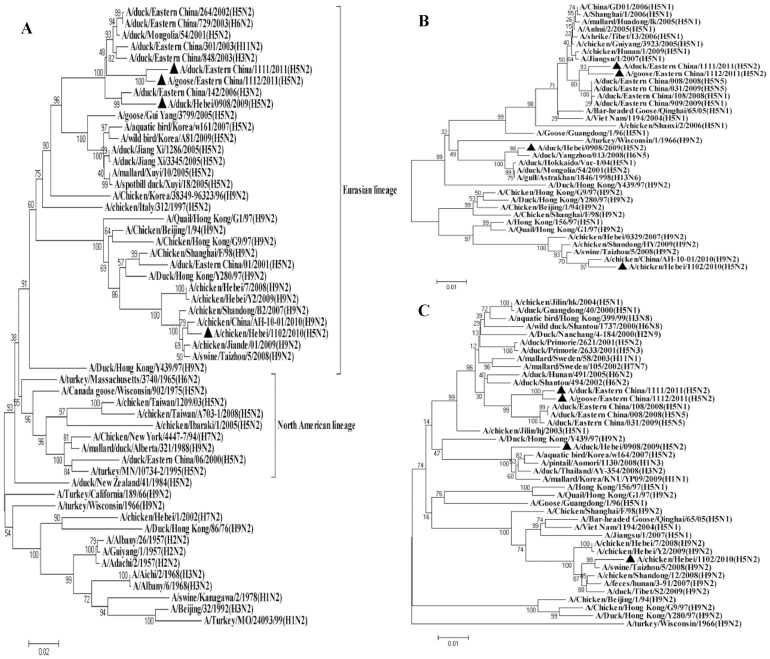
Phylogenetic trees based on the open reading frame sequences of neuraminidase (A), matrix protein (B) and nucleocapsid protein (C) genes of H5N2 viruses in this study and those of reference strains from GenBank. Viruses highlighted with a closed triangle were those characterised in this study. The trees were constructed using the neighbour-joining method of MEGA 4.0 with 1,000 bootstrap trials performed to assign confidence to the grouping.

**Figure 3:**
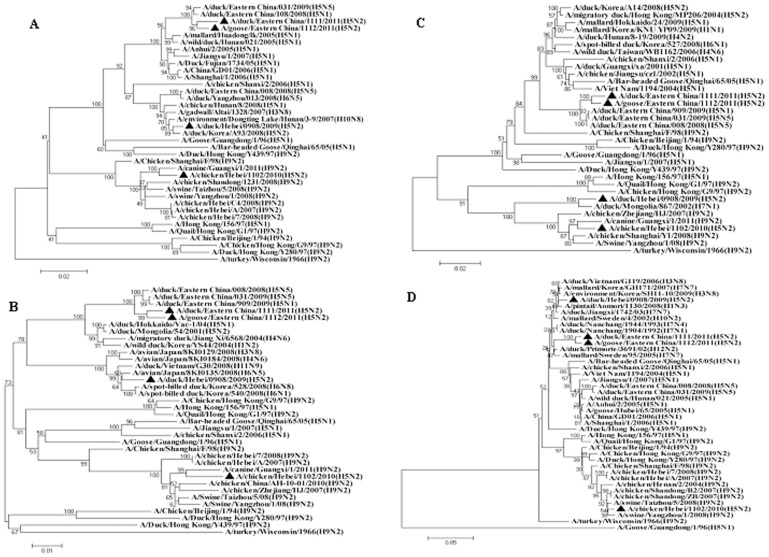
Phylogenetic trees based on the open reading frame sequences of polymerase acidic protein (A), polybasic protein 1 (B), polybasic protein 2 (C) and nonstructural protein (D) genes of H5N2 viruses in this study and those of reference strains from GenBank.

To explore the origination of these H5N2 viruses, the whole genome of each virus was sequenced and phylogenetically analysed together with a number of gene sequences selected from the GenBank. The analysis revealed that these novel H5N2 viruses were likely originated from the reassortment between HPAI H5N1 and viruses of other subtypes. In the HA tree, DK/EC/1111/11 and GS/EC/1112/1 clustered with H5N1 viruses of clade 2.3.4, both isolates shared 96.8%∼97.0% nucleotide homology with a clade 2.3.4 virus A/duck/Eastern China/108/2008. The other two isolates, CK/HB/1102/10 and DK/HB/0908/09, fell into clade 7, with 97.0% homology to H5N1 virus A/chicken/Vietnam/NCVD-swab15/2008 and 99.0% to H5N1 virus A/chicken/Huadong/4/2008, respectively (Table 2). These data suggest the H5N2 viruses have acquired their HA genes from different clades of H5N1 viruses. All of the 4 H5N2 isolates clustered with Eurasian viruses rather than North American ones in the NA tree. Although DK/HB/0908/09 was isolated in ducks from Hebei, this virus and the two eastern Chinese H5N2 viruses clustered in the tree with a duck H3N2 virus A/duck/Eastern China/142/2006, while another Hebei isolate, CK/HB/1102/10, was closely related with H9N2 viruses (Fig. 2A). However, a close examination on the internal gene trees (Fig. 2 and 3) and genetic similarity (Table 2) revealed that the Hebei duck isolate DK/HB/0908/09 has a more diversified origin of internal genes than the Hebei chicken isolate CK/HB/1102/10 and the two Eastern Chinese H5N2 viruses. For CK/HB/1102/10, the internal genes shared the highest genetic identities to either A/Chicken/Shanghai/F/98-like viruses or A/Quail/Hong Kong/G1/97-like H9N2 viruses, suggesting an H9N2 origin of the internal genes. All internal genes of DK/EC/1111/11 and GS/EC/1112/11 were closely related to those of H5N1 viruses (A/duck/Eastern China/909/2009 and A/duck/Eastern China/108/2008), except the non-structural protein (NS) gene. The NS gene of both viruses has high genetic similarity (95.9%∼96.0%) to an H7N1 virus A/duck/Nanchang/1904/1992 virus. In the case of DK/HB/0908/09 virus, the polymerase basic protein 2 (PB2) gene was closely related to an H7N1 virus, while its polymerase basic protein 1 (PB1) and matrix protein (M) genes were almost identical to those of H6N5 viruses, e.g. A/avian/Japan/8KI0135/2008 and A/duck/Yangzhou/013/2008. The polymerase acidic protein (PA) and nucleoprotein (NP) genes of this virus, however, were highly similar to those of Korean H5N2 isolates. In addition, its NS gene was genetically very close to a Korean H3N8 virus. Taken together, it seems that the Eastern Chinese H5N2 isolates DK/EC/1111/11 and GS/EC/1112/11 were generated from reassortment events in which the NA and NS genes of HPAI H5N1 viruses were replaced with those of other influenza subtypes. For the two Heibei viruses, while the HA gene of both viruses was from H5N1 viruses, CK/HB/1102/10 probably derived its internal genes from endemic H9N2 viruses of A/Chicken/Shanghai/F/98-like and A/Quail/Hong Kong/G1/97-like, and those of DK/HB/0908/09 were from various subtypes (Table 2).

**Table 2:** Influenza viruses with the highest nucleotide identity to each gene of A/duck/Eastern China/1111/2011, A/chicken/Hebei/1102/2010 and A/duck/Hebei/0908/2009*.

Genesegment	A/duck/Eastern China/1111/2011		A/chicken/Hebei/1102/2010		A/duck/Hebei/0908/2009	
	Closest viruses	Nucleotide identity	Closest viruses	Nucleotide identity	Closest viruses	Nucleotide identity
PB2	DK/EC/909/09 (H5N1)	98%	CK/SH/Y1/08 (H9N2)	98%	DK/MG/867/02 (H7N1)	98%
PB1	DK/EC/909/09 (H5N1)	98%	CK/China/AH-10-01/10 (H9N2)	97%	JP/8KI0135/08 (H6N5)	99%
PA	DK/EC/108/08 (H5N1)	98%	CK/SD/1231/08 (H9N2)	97%	DK/Korea/A93/08 (H5N2)	99%
HA	DK/EC/108/08 (H5N1)	97%	CK/VN/NCVD-swab15/08 (H5N1)	97%	CK/HD/4/08 (H5N1)	99%
NP	DK/EC/108/08 (H5N1)	97%	CK/SD/12/08 (H9N2)	97%	AB/Korea/w164/07 (H5N2)	97%
NA	DK/EC/142/06 (H3N2)	95%	CK/China/AH-10-01/10 (H9N2)	98%	DK/EC/142/06 (H3N2)	97%
M	DK/EC/909/09 (H5N1)	98%	CK/China/AH-10-01/10 (H9N2)	98%	DK/YZ/013/08 (H6N5)	99%
NS	DK/NC/1904/92 (H7N1)	96%	CK/SD/ZB/07 (H9N2)	98%	Korea/SH11-10/09 (H3N8)	98%

* Abbreviations: PB, polybasic protein; PA, polymerase acidic protein; HA, haemagglutinin; NP, nucleocapsid protein; NA, neuraminidase; M, matrix protein; NS, nonstructural protein; DK/EC/909/09, A/duck/Eastern China/909/2009; DK/EC/108/08, A/duck/Eastern China/108/2008; DK/EC/142/06, A/duck/Eastern China/142/2006; DK/NC/1904/92, A/duck/Nanchang/1904/1992; CK/SH/Y1/08, A/chicken/Shanghai/Y1/2008; CK/China/AH-10-01/10, A/chicken/China/AH-10-01/2010; CK/SD/1231/08, A/chicken/Shandong/1231/2008; CK/VN/NCVD-swab15/08, A/chicken/Vietnam/NCVD-swab15/2008; CK/SD/12/08, A/chicken/Shandong/12/2008; CK/SD/ZB/07, A/chicken/Shandong/ZB/2007; DK/MG/867/02, A/duck/Mongolia/867/2002; JP/8KI0135/08, A/avian/Japan/8KI0135/2008; DK/Korea/A93/08, A/duck/Korea/A93/2008; CK/HD/4/08, A/chicken/Huadong/4/2008; AB/Korea/w164/07, A/aquatic bird/Korea/w164/2007; DK/EC/142/06, A/duck/Eastern China/142/2006; DK/YZ/013/08, A/duck/Yangzhou/013/2008; Korea/SH11-10/09, A/environment/Korea/SH11-10/2009. Data for A/goose/Eastern China/1112/2011 were idential to those for A/duck/Eastern China/1111/2011 and were not shown in this table.

### Molecular Characteristics of Viral Genes

The HA gene of both DK/HB/0908/09 and CK/HB/1102/10 has an open reading frame (ORF) of 1,707 nucleotides (nt), coding for 569 amino acids, while that of DK/EC/1111/11 and GS/EC/1112/11 has an ORF of 1,704 nt, coding for 568 amino acid residues, including a signal peptide of 16 amino acids long. All of the H5N2 isolates possessed amino acid sequence characteristic of HPAI viruses (Table 1) at the cleavage site between HA1 and HA2 [13]. BLAST (http://blast.ncbi.nlm.nih.gov/) analysis revealed that DK/EC/1111/11 and GS/EC/1112/11 shared the highest HA nucleotide sequence similarity with H5N1 viruses isolated in humans from 2005–2006 in China [14], [15], although the HA cleavage motif in these two viruses (PLREKRR-KR/G or PLRGKRR-KR/G) was different from that found in human strains (PLRERRRKR/GL). Analysis on the consensus amino acid sequence of DK/EC/1111/11 and GS/EC/1112/11 revealed 4 potential N-linked glycosylation sites in HA1 (20 or 21, 33, 169 and 289) and two in HA2 (154 and 212) (H3 numbering), similar to clade 2.3.4 H5N1 viruses isolated from humans between 2005 and 2006. The consensus amino acid sequence of DK/HB/0908/09 and CK/HB/1102/10 has 6 potential N-linked glycosylation sites in HA1 (20 or 21, 33, 81, 158, 167 and 289) and two in HA2 (154 and 212), similar to clade 7 viruses. In addition, CK/HB/1102/10 has 2 potential N-linked glycosylation sites in HA1 (276 and 240). The conservative residues within the receptor binding pocket of the HA, including E190, R220, G225, Q226 and G228, were all present in these viruses, implying that they retain a typical avian virus-like receptor specificity [16]. Previous studies have shown that there are possibly 5 antigenic epitopes in HA1 associated with amino acid residues at positions 46, 62, 122, 126, 129–134, 140–145, 155–157, 160, 166, 186 and 187 [16], [17], [18], [19]. An examination on these positions revealed R62K, A132T, S134L and T160A substitutions in HA1 of DK/EC/1111/11 and GS/EC/1112/11, and E131D, A132T and G143D in HA1 of CK/HB/1102/10.

Compared with DK/HB/0908/09, DK/EC/1111/11 and GS/EC/1112/11, the NA of CK/HB/1102/10 had a deletion of 9 residues in the stalk region, resulting in the loss of a potential glycosylation site. None of the 4 H5N2 viruses carries the Q274Y mutation in the NA, which is associated with oseltamivir resistance [20]. However, the matrix protein (M) of these viruses, except that of DK/HB/0908/09, has the S31N substitution, which is associated with amantadine resistance [20]. In the polymerase basic protein 2 (PB2), these viruses possess E and D at 627 and 701, two positions that are related to the pathogenicity of influenza viruses in the mammalian model [21], [22].

### Pathogenicity Experiments

The World Organisation for Animal Health (OIE) recommended that H5 HPAI virus has an IVPI greater than 1.2 or possesses characteristic multibasic amino acid sequences of HPAI virus at the cleavage site of HA (http://www.oie.int/en/international-standard-setting/terrestrial-manual/access-online/). Besides, based on the 50% mouse lethal dose (MLD_50_), a couple of previous publications established that influenza viruses can be classified as low (MLD_50_>6.5 log_10_EID_50_), medium (3 log_10_EID_50_< MLD_50_≤6.5 log_10_EID_50_), or high (MLD_50_≤3 log_10_EID_50_) pathogenic to mice [23]. To examine the pathogenicity of these H5N2 viruses in poultry and mammalian models, we measured the intravenous pathogenicity index (IVPI) in chickens and ducks, and the MLD_50_ in BALB/c mice for each virus. DK/EC/1111/11 and GS/EC/1112/11 were highly pathogenic for chickens and ducks by the IVPI criteria and moderately pathogenic for mice by the MLD_50_ criteria [23], [24], CK/HB/1102/10 was only highly pathogenic for chickens, whereas DK/HB/0908/09 was low pathogenic for all three species (Table 1).

### Replication and Transmission in Ducks

**Figure 4:**
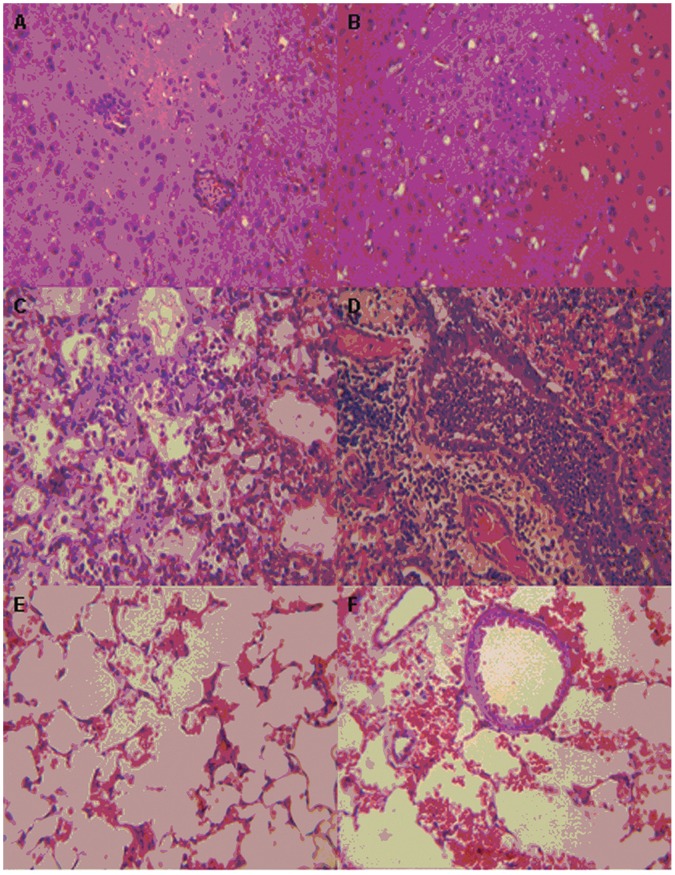
Representative photomicrographs of hematoxylin-andeosin-stained tissues from ducks and mice inoculated with H5N2 viruses. (A) Perivascular lymphoplasmacytic cuffs around a few cerebral vessels in the cerebrum of a mallard duck infected with DK/EC/1111/11 and euthanatized on 5 dpi. (B) Small foci of gliosis in the cerebrum of a mallard duck infected with GS/EC/1112/11 and euthanatized on 5 dpi. (C and D) Mild pneumonorrhagia in the lung of BALB/c mice infected with DK/HB/0908/09 (C) and CK/HB/1102/10 (D), on 5 dpi. (E and F) Mild to severe pneumonorrhagia with lymphohistiocytic alveolitis in the lung of BALB/c mice infected with DK/EC/1111/11 (E) and GS/EC/1112/11 (F), on 5 dpi.

Since ducks are usually in contact with chickens and wild migratory birds, we examined the replication and transmission of these H5N2 viruses in domestic ducks. Ducks infected with DK/EC/1111/11 and GS/EC/1112/11 showed clinical symptoms such as coughing, ocular and nasal discharge and conjunctivitis between 3 and 7 day-post-inoculation (dpi). Gross lesions typical of HPAI virus infection, including haemorrhage and necrosis in multiple organs, were observed in ducks euthanatized on 3 and 5 dpi. Virus was detected in all tested visceral organs (heart, liver, spleen, lung, kidney and brain) and skeletal muscle from these euthanatized birds (Table 3), and in tracheal and cloacal swabs from inoculated birds (Table 4). Virus was also detected in tracheal and cloacal swabs collected from contact birds between 2 and 7 dpi (Table 4). These data indicated systemic infection by DK/EC/1111/11 and GS/EC/1112/11 and the transmissibility of these two viruses among domestic ducks. In contrast, ducks inoculated with DK/HB/0908/09 and CK/HB/1102/10 did not show noticeable clinical symptoms. Nevertheless, the inoculated birds shed virus for 3–5 days and both viruses were transmitted to the naïve cage mates via direct contact. Histologic lesions were observed in ducks inoculated each of the 4 H5N2 viruses, however lesions were more severe in ducks inoculated with either DK/EC/1111/11 or GS/EC/1112/11. The lesions included multifocal necrosis in various visceral organs, and myocarditis, hepatitis, splenitis, interstitial pneumonia, nephritis and hyalinization to necrosis of skeletal myofibers. Moderate lesions, characterized by perivascular lymphoplasmacytic cuffs around a few localized cerebral vessels and associated small foci of gliosis (Fig. 4A and 4B), were also observed in the brain of infected ducks. In contrast, ducks inoculated with DK/HB/0908/09 or CK/HB/1102/10 virus showed less evident hispathological changes, including mild multifocal hepatitis, splenitis, interstitial pneumonia and nephritis.

**Table 3:** Virus titers in various tissues of mallard ducks infected with H5N2 viruses.

Strain	Day post-inoculation	Virus titres (log_10_EID_50_ g^-1^ tissue) in						
		Heart	Liver	Spleen	Lung	Kidney	Brain	skeletal muscle
DK/HB/0908/09	3	0/2	0/2	0/2	2/2(1.3±0.1)	0/2	0/2	0/2
	5	0/2	0/2	0/2	0/2	0/2	0/2	0/2
CK/HB/1102/10	3	0/2	0/2	0/2	2/2 (2.1±0.2)	0/2	0/2	0/2
	5	0/2	0/2	0/2	0/2	0/2	0/2	0/2
DK/EC/1111/11	3	2/2 (3.8±0.7)	2/2 (3.0±0.5)	2/2 (3.2±0.5)	2/2 (4.5±0.2)	2/2 (4.0±0.5)	2/2 (2.8±0.5)	2/2 (3.5±0.2)
	5	2/2 (5.2±0.7)	2/2 (2.4±0.1)	2/2 (<1)	2/2 (4.6±0.3)	2/2 (3.1±0.5)	2/2 (5.4±0.3)	2/2 (3.0±0.6)
GS/EC/1112/11	3	2/2 (5.0±0.3)	2/2 (2.6±0.3)	2/2 (3.1±0.4)	2/2 (4.0±0.7)	2/2 (4.3±0.1)	2/2 (2.1±0.2)	2/2 (3.2±0.2)
	5	2/2 (5.4±0.3)	2/2 (2.3±0.2)	1/2 (<1)	2/2 (4.4±0.1)	2/2 (3.3±0.2)	2/2 (1.5±0.2)	2/2 (3.1±0.1)

a Virus positive birds/tested birds.

b Average virus titer of infected samples (log_10_ EID_50_±SD).

**Table 4:** Virus titers in oropharyngeal and cloacal swabs of experimentally infected mallards.

Strain	Infection sample	2 dpi		3 dpi		5 dpi		7 dpi		9 dpi	
		T	C	T	C	T	C	T	C	T	C
DK/HB/0908/09	Inoculated	4/4^a^ (1.3±0.2)^b^	4/4 (1.2±0.1)	4/4 (1.6±0.2)	4/4 (2.3±0.1)	4/4 (1.2±0.2)	4/4 (1.3±0.2)	3/4 (<1)	2/4 (<1)	0/4	0/4
	Contacted	0/2	0/2	2/2 (1.5±0.2)	2/2 (2.4±0.1)	2/2 (1.3±0.1)	2/2 (1.1±0.1)	1/2 (<1)	2/2 (<1)	0/2	0/2
CK/HB/1102/10	Inoculated	4/4 (1.3±0.2)	4/4 (1.4±0.1)	4/4 (2.3±0.4)	4/4 (2.3±0.2)	4/4 (1.9±0.4)	4/4 (1.6±0.4)	3/4 (<1)	1/4 (<1)	0/4	0/4
	Contacted	0/2	0/2	2/2 (2.4±0.1)	2/2 (2.6±0.1)	2/2 (1.6±0.3)	2/2 (1.3±0.2)	2/2 (<1)	1/2 (<1)	0/2	0/2
DK/EC/1111/11	Inoculated	4/4 (2.5±0.7)	4/4 (1.8±0.4)	4/4 (3.4±0.9)	4/4 (1.9±0.4)	4/4 (2.4±0.7)	4/4 (1.5±0.4)	3/4 (<1)	2/4 (<1)	0/4	0/4
	Contacted	2/2 (1.4±0.3)	2/2 (1.7±0.2)	2/2 (4.0±0.2)	2/2 (1.4±0.1)	2/2 (2.0±0.5)	2/2 (1.3±0.2)	2/2 (<1)	2/2 (<1)	0/2	0/2
GS/EC/1112/11	Inoculated	4/4 (3.9±0.9)	4/4 (1.9±0.5)	4/4 (4.2±0.3)	4/4 (3.5±0.1)	4/4 (1.2±0.2)	4/4 (1.3±0.7)	3/4 (<1)	4/4 (<1)	0/4	0/4
	Contacted	2/2 (2.2±0.5)	2/2 (1.8±0.1)	2/2 (2.4±0.2)	2/2 (1.8±0.5)	2/2 (2.0±0.2)	2/2 (1.3±0.2)	2/2 (<1)	2/2 (<1)	0/2	0/2

Abbreviations: dpi, day post-inoculation; T, oropharyngeal swab; C, cloacal swab.

a Virus positive birds/tested birds.

b Average virus titer of infected samples (log_10_ EID_50_±SD).

### Replication in Mice

To investigate the replication of these H5N2 viruses in the mouse model, BALB/c mice (6-week-old, female) were inoculated intranasally with each virus, and the viral load in various organs was titrated. All of the 4 H5N2 isolates were recovered only in the lung, with mean titres (log_10_ EID_50_) of 3.5±0.5, 3.8±0.4, 3.1±0.4, 2.5±0.2 on three dpi, and 3.4±0.4, 3.5±0.2, 2.3±0.2, 2.3±0.4 on 5 dpi, for DK/EC/1111/11, GS/EC/1112/11, CK/HB/1102/10 and DK/HB/0908/09 respectively. Histologic lesions observed in mice inoculated with either DK/EC/1111/11 or GS/EC/1112/11 was more evident. Tracheitis, severe acute necrotizing bronchitis and moderate alveolitis were present (Fig. 4C and 4D). By contrast, only mild pneumonorrhagia was observed in mice inoculated with either DK/HB/0908/09 or CK/HB/1102/10 (Fig. 4E and 4F). Taken together, these results demonstrated that although DK/HB/0908/09 and CK/HB/1102/10 replicated efficiently in respiratory tissues without any adaptation, both viruses were less pathogenic to BALB/c mice than DK/EC/1111/11 and GS/EC/1112/11.

## Discussion

The Asian HPAI H5N1 viruses that emerged over a decade ago in China have evolved into over 10 distinct phylogenetic clades [1], however, all Asian HPAI viruses containing H5 matched only with the N1 subtype. Since 2000, H5N1 viruses have been involved in various reassortment events, contributing genetic components to influenza viruses of other subtypes, or resulting in novel H5 viruses with an NA other than the N1 subtype, such as H5N5 [2], [4], [25], [26]. In this study, two HPAI H5N2 viruses, DK/EC/1111/11, and GS/EC/1112/11, whose HA gene fell into clade 2.3.4 in the H5 phylogenetic tree, were isolated from ducks and geese from the same geographical location as where the H5N5 viruses were detected. There might be multiple reasons why HPAI H5N5 and H5N2 viruses were isolated from the same place. One of these is the long term endemicity of H5N1 HPAI viruses in poultry and the common practice of intermingling raising of chickens and domestic ducks and geese, which potentially provides an ideal environment for the generation of reassortant H5 HPAI viruses with NA subtypes other than N1. Considering the endemicity of clade 2.3.4 subtype H5N1 viruses in China since 2005 [1], it is plausible that H5N1 viruses have provided the backbone for generating these two novel H5N2 viruses rather than gene flow in the opposite direction. It is important to recognise that mass vaccination is the main strategy for the control of H5N1 HPAI in poultry in China [27]; however vaccination alone can never eliminate influenza virus in poultry flocks, especially in domestic waterfowl. The emergence of these novel reassortant viruses in apparently healthy domestic poultry in China indicates that the HPAI H5 virus probably cannot be stopped without more comprehensive control measures.

Our phylogenetic analysis suggested that CK/HB/1102/10, a chicken H5N2 virus isolated from Hebei Province, China, bears HA gene from clade 7 H5N1 virus. The remaining 7 genes of this virus, however, are likely from A/Chicken/Shanghai/F/98-like and A/Quail/Hong Kong/G1/97-like H9N2 viruses (Fig. 2 and 3). H9N2 viruses are present worldwide in poultry and derive from two major influenza virus gene pools, the Eurasian and the North American [28], [29]. In China, H9N2 influenza viruses have been circulating since 1994, and major genotypes were divided into five series [30]. The A/Quail/Hong Kong/G1/97-like viruses have been postulated to provide internal genes to H5N1 viruses isolated from Hong Kong in 1997 [31]. Our findings further validate that H9N2 and H5N1 subtype viruses have a two-way exchange of gene segments to generate current genotypes of both subtypes that have pandemic potential [32].

Our studies indicated that 3 of the 4 H5N2 isolates inherited highly pathogenic phenotype from the reassortment involved HPAI H5N1 viruses. This is different from HPAI H5N2 viruses reported in America, which acquired high pathogenicity through accumulation of mutations in viral components, particularly the HA, after adaptation in poultry [8]. HPAI H5N2 viruses have been detected in wild waterfowl in Africa [33]. These African H5N2 viruses were unrelated to any strains of HPAI H5N1 viruses. Instead, have close relationships with H5 viruses of low pathogenicity circulating in Eurasian wild and domestic ducks. The emergence of HPAI H5N2 viruses through reassortment in Asia further highlighted the special etiology of influenza in this area and the active interaction of H5N1 strains with viruses of other subtypes.

It is worthy of noting that one H5N2 isolate in this study, DK/HB/0908/09, was low pathogenic to chickens, ducks and mice, albeit the presence of cleavage motif characteristic of HPAI virus. The low pathogenic phenotype of H5 viruses with genetic coding for highly pathogenic viruses has been recorded previously [34]. The mechanisms that prevent the highly pathogenic potential have been attributed to an additional glycosylation site in HA, or amino acid mutation at position 149 of NS1 protein. A close check of DK/HB/0908/09 virus suggested that neither of these mechanisms applies to its low pathogenicity. This fact adds to the concept that the amino acid sequence at the HA cleavage site is not the only genetic determinant for the high pathogenicity of H5N2 influenza viruses. More work remains to be done to elucidate the lack of highly pathogenicity of H5N2 viruses such as DK/HB/0908/09.

In contrast to those low pathogenic H5N2 viruses previously reported in Asia, some of which even failed to replicate in experimental chickens [35], H5N2 viruses characterized in the present study readily replicated in mice without prior adaptation. More experiments should be performed, particularly, in mammalian models, to evaluate the potential public threat posed by these novel reassortant H5N2 viruses.

In conclusion, all H5N2 isolates characterized in this study grouped in the HA tree with the Asian HPAI H5N1 viruses and none of these isolates grouped with those low pathogenic H5N2 viruses previously reported in Asia [9], [10], [11], [12]. In view of the endemicity that the Asian HPAI H5N1 viruses have gained in China since 1996 and widespread use of avian influenza vaccines, the presence of reassortant H5N2 avian influenza viruses in apparently healthy domestic ducks, geese and chickens highlights that domestic poultry could serve as reassortant vessels for creating new different genetic backgrounds of the HPAI. Despite all the H5N2 isolates in this study possessed the characteristic sequence of HPAI virus at the cleavage site between the HA1 and HA2 proteins, animal experiments indicated that they have different pathogenicities to chickens, domestic ducks and mice. It is clear that the HPAI H5 virus originated from outbreaks in poultry, and its geographical spread and significant viral reassortment events is unlikely to be stopped without the implementation of more comprehensive control measures in the global poultry industry as well as wild birds.

## Materials and Methods

### Ethics Statement

All animal research was approved by the Jiangsu Administrative Committee for Laboratory Animals (Permission number: SYXK-SU-2007-0005), and complied with the guidelines of Jiangsu laboratory animal welfare and ethical of Jiangsu Administrative Committee of Laboratory Animals.

### Virus Isolation and Identification

As part of routine surveillance on avian influenza viruses, we performed a monthly sampling in a LBM in Yangzhou, Jiangsu Province, China, from July 2002 to May 2011. This LBM is the largest one in Eastern China, and has a total daily transaction of 10,000 geese and 50,000 chickens, 5,000 ducks. Cloacal swabs were collected from 5 chickens, 15 ducks and 15 geese randomly selected from each consignment shipped to the LBM from local farms or introduced from neighbouring provinces, such as Shandong, Anhui, Zhejiang and Hebei. Swab samples were maintained in transport medium containing antibiotics and kept at 4°C until transported to the laboratory. Samples were processed and inoculated into embryonated specific pathogen-free (SPF) chicken eggs. The presence of virus in allantoic fluid was confirmed with hemagglutination assay. Viruses were plaque purified three times in Madin-Darby canine kidney (MDCK) cells and propagated in eggs. The HA and NA subtypes were identified with reverse transcription-polymerase chain reaction (RT-PCR) and sequencing as previously described [36], [37]. All experiments with infectious virus were conducted in biosafety level 3 laboratory facilities.

### Viral Sequencing

Viral RNA was extracted from allantoic fluid with Trizol LS reagent (Invitrogen, Carlsbad, CA). Viral RNA was reverse transcribed with the 12 bp primer 5′-AGCAAAAGCAGG-3′. PCR was performed using specific primers as described by Hoffmann et al. [38]. PCR products were purified with the TaKaRa Agarose Gel DNA Purification Kit Ver. 2.0 (TaKaRa, Dalian, China) and sequenced by the Nanjing GenScript Biotech Co., Ltd.

### Phylogenetic Analyses

Influenza virus sequences used in the phylogenetic comparison in this study were obtained from the NCBI Influenza Virus Resource (http://www.ncbi.nlm.nih.gov/genomes/FLU/). The representative sequences were selected by randomly choosing one that had been isolated from China and that was similar to other sequences (genetic distance <2.0%). Some representative sequences from other countries were also included in the analysis. Editing, analysis and alignment of sequence data were performed with BioEdit 7.0 and Clustal X, respectively. Phylogenetic trees based on coding sequences of individual genes were constructed using the Kimura two-parameter model and neighbour-joining algorithm in the program MEGA (version 4.0) with 1,000 bootstraps. All branches supported by a >70% bootstrap value were considered as the same group in the phylogenetic trees. The sequence data obtained in this study are available in GenBank under accession numbers JQ041387∼JQ041418.

### Animal Experiments

In the IVPI test, 4 groups of ten 6-week-old SPF chickens (Beijing Experimental Animal Centre, Beijing) and 4 groups of ten 4-week-old mallard ducks (anas platyhynchos) were intravenously inoculated with 0.2 ml of 1∶10 dilution of the infectious allantoic fluid of each H5N2 isolate. Oropharyngeal and cloacal swabs, and sera from each duck were collected for viral isolation and antibody detection to exclude pre-existing influenza A virus infection. The EID_50_ was determined by serial titration of virus in SPF eggs and was calculated with the method described by Reed and Muench [39]. Groups of 6-week-old female BALB/c mice (Beijing Experimental Animal Centre, Beijing) were lightly anesthetized and inoculated intranasally with 10^0^ to 10^7^ EID_50_ of each virus in diluted in 50 µl PBS to evaluate the MLD_50_ [39], [40].

To examine the replication and transmission of these H5N2 viruses in ducks, 4 groups of 4-week-old mallard ducks (eight birds/group) were intranasally inoculated with 10^3.0^ EID_50_ of each virus in a volume of 0.1 ml. On 1 dpi, another eight 4-week-old mallard ducks (2 for each group) were placed into the same isolator to serve as contacts. Ducks were observed daily for clinical signs of disease. On 2, 3, 5, 7, 9, 11 and 14 dpi, oropharyngeal and cloacal swabs were collected from 4 inoculated ducks and 2 contact birds to estimate the virus shedding. In addition, on 3 and 5 dpi, 4 inoculated ducks were euthanatized and tissues (heart, liver, spleen, lung, kidney, brain and skeletal muscle) were collected from each duck for virus titration, observation of gross lesions and histopathology [41].

To investigate the replication of each virus in mice, 4 groups of nine 6-week-old female BALB/c mice were inoculated intranasally with 10^5.0^ EID_50_ of each virus in 50 µl PBS under slight anaesthesia. Control mice were mock-infected with PBS. Three mice per group were euthanatized on 3 and 5 dpi, and organs (heart, liver, spleen, lung, kidney and brain) were harvested for virus isolation. Briefly, tissues were homogenised in 1.0 ml PBS. After clarification, the supernatant was titrated in embryonated SPF eggs for infectivity. In addition, 3 mice per group were euthanatized at 5 dpi, and organs (heart, liver, spleen, lung, kidney and brain) were collected for histopathology [41].

All animal experiments were performed in animal biosafety level 3 facilities at Yangzhou University.

## References

[1] Eagles D, Siregar ES, Dung DH, Weaver J, Wong F, et al (2009) H5N1 highly pathogenic avian influenza in Southeast Asia. Rev Sci Tech 28: 341–348. 19618637 10.20506/rst.28.1.1864

[2] Guan Y, Peiris JS, Lipatov AS, Ellis TM, Dyrting KC, et al (2002) Emergence of multiple genotypes of H5N1 avian influenza viruses in Hong Kong SAR. Proc Natl Acad Sci U S A 99: 8950–8955. 12077307 10.1073/pnas.132268999 PMC124404

[3] Guan Y, Poon LL, Cheung CY, Ellis TM, Lim W, et al (2004) H5N1 influenza: a protean pandemic threat. Proc Natl Acad Sci U S A 101: 8156–8161. 15148370 10.1073/pnas.0402443101 PMC419573

[4] Gu M, Liu W, Cao Y, Peng D, Wang X, et al (2011) Novel Reassortant Highly Pathogenic Avian Influenza (H5N5) Viruses in Domestic Ducks, China. Emerg Infect Dis 17: 1060–1063. 21749770 10.3201/eid1706.101406 PMC3358203

[5] Li KS, Guan Y, Wang J, Smith GJ, Xu KM, et al (2004) Genesis of a highly pathogenic and potentially pandemic H5N1 influenza virus in eastern Asia. Nature 430: 209–213. 15241415 10.1038/nature02746

[6] Chen H, Smith GJ, Li KS, Wang J, Fan XH, et al (2006) Establishment of multiple sublineages of H5N1 influenza virus in Asia: implications for pandemic control. Proc Natl Acad Sci U S A 103: 2845–2850. 16473931 10.1073/pnas.0511120103 PMC1413830

[7] Snoeck CJ, Adeyanju AT, De Landtsheer S, Ottosson U, Manu S, et al (2011) Reassortant low-pathogenic avian influenza H5N2 viruses in African wild birds. J Gen Virol 92: 1172–1183. 21248176 10.1099/vir.0.029728-0

[8] Garcia M, Crawford JM, Latimer JW, Rivera-Cruz E, Perdue ML (1996) Heterogeneity in the haemagglutinin gene and emergence of the highly pathogenic phenotype among recent H5N2 avian influenza viruses from Mexico. J Gen Virol 77 (Pt 7): 1493–1504. 10.1099/0022-1317-77-7-1493 8757992

[9] Cheng MC, Soda K, Lee MS, Lee SH, Sakoda Y, et al (2010) Isolation and characterization of potentially pathogenic H5N2 influenza virus from a chicken in Taiwan in 2008. Avian Dis 54: 885–893. 20608534 10.1637/9208-120609-Reg.1

[10] Lee JH, Pascua PN, Song MS, Baek YH, Kim CJ, et al (2009) Isolation and genetic characterization of H5N2 influenza viruses from pigs in Korea. J Virol 83: 4205–4215. 19359528 10.1128/JVI.02403-08 PMC2668473

[11] Guang-Jian Z, Zong-Shuai L, Yan-Li Z, Shi-Jin J, Zhi-Jing X (2011) Genetic characterization of a novel influenza A virus H5N2 isolated from a dog in China. Vet Microbiol 155(2–4): 409–16. 22033043 10.1016/j.vetmic.2011.08.017

[12] Ogata T, Yamazaki Y, Okabe N, Nakamura Y, Tashiro M, et al (2008) Human H5N2 avian influenza infection in Japan and the factors associated with high H5N2-neutralizing antibody titer. J Epidemiol 18: 160–166. 18603824 10.2188/jea.JE2007446 PMC4771585

[13] Stieneke-Grober A, Vey M, Angliker H, Shaw E, Thomas G, et al (1992) Influenza virus hemagglutinin with multibasic cleavage site is activated by furin, a subtilisin-like endoprotease. EMBO J 11: 2407–2414. 1628614 10.1002/j.1460-2075.1992.tb05305.x PMC556715

[14] Sun L, Lu X, Li C, Wang M, Liu Q, et al (2009) Generation, characterization and epitope mapping of two neutralizing and protective human recombinant antibodies against influenza A H5N1 viruses. PLoS One 4: e5476. 19421326 10.1371/journal.pone.0005476 PMC2674214

[15] Li Y, Shi J, Zhong G, Deng G, Tian G, et al (2010) Continued evolution of H5N1 influenza viruses in wild birds, domestic poultry and humans in China from 2004 to 2009. J Virol 84(17): 8389–97. 20538856 10.1128/JVI.00413-10 PMC2919039

[16] Philpott M, Hioe C, Sheerar M, Hinshaw VS (1990) Hemagglutinin mutations related to attenuation and altered cell tropism of a virulent avian influenza A virus. J Virol 64: 2941–2947. 2335822 10.1128/jvi.64.6.2941-2947.1990 PMC249478

[17] Philpott M, Easterday BC, Hinshaw VS (1989) Neutralizing epitopes of the H5 hemagglutinin from a virulent avian influenza virus and their relationship to pathogenicity. J Virol 63: 3453–3458. 2473218 10.1128/jvi.63.8.3453-3458.1989 PMC250921

[18] Kaverin NV, Rudneva IA, Ilyushina NA, Varich NL, Lipatov AS, et al (2002) Structure of antigenic sites on the haemagglutinin molecule of H5 avian influenza virus and phenotypic variation of escape mutants. J Gen Virol 83: 2497–2505. 12237433 10.1099/0022-1317-83-10-2497

[19] Kaverin NV, Rudneva IA, Govorkova EA, Timofeeva TA, Shilov AA, et al (2007) Epitope mapping of the hemagglutinin molecule of a highly pathogenic H5N1 influenza virus by using monoclonal antibodies. J Virol 81: 12911–12917. 17881439 10.1128/JVI.01522-07 PMC2169086

[20] Scholtissek C, Quack G, Klenk HD, Webster RG (1998) How to overcome resistance of influenza A viruses against adamantane derivatives. Antiviral Res 37: 83–95. 9588841 10.1016/s0166-3542(97)00061-2

[21] Li Z, Chen H, Jiao P, Deng G, Tian G, et al (2005) Molecular basis of replication of duck H5N1 influenza viruses in a mammalian mouse model. J Virol 79: 12058–12064. 16140781 10.1128/JVI.79.18.12058-12064.2005 PMC1212590

[22] Hatta M, Gao P, Halfmann P, Kawaoka Y (2001) Molecular basis for high virulence of Hong Kong H5N1 influenza A viruses. Science 293: 1840–1842. 11546875 10.1126/science.1062882

[23] Chen H, Deng G, Li Z, Tian G, Li Y, et al (2004) The evolution of H5N1 influenza viruses in ducks in southern China. Proc Natl Acad Sci U S A 101: 10452–10457. 15235128 10.1073/pnas.0403212101 PMC478602

[24] Katz JM, Lu X, Tumpey TM, Smith CB, Shaw MW, et al (2000) Molecular correlates of influenza A H5N1 virus pathogenesis in mice. J Virol 74: 10807–10810. 11044127 10.1128/jvi.74.22.10807-10810.2000 PMC110957

[25] Cong YL, Pu J, Liu QF, Wang S, Zhang GZ, et al (2007) Antigenic and genetic characterization of H9N2 swine influenza viruses in China. J Gen Virol 88: 2035–2041. 17554038 10.1099/vir.0.82783-0

[26] Xu C, Fan W, Wei R, Zhao H (2004) Isolation and identification of swine influenza recombinant A/Swine/Shandong/1/2003(H9N2) virus. Microbes Infect 6: 919–925. 15310468 10.1016/j.micinf.2004.04.015

[27] Chen H (2009) Avian influenza vaccination: the experience in China. Rev Sci Tech 28: 267–274. 19618631 10.20506/rst.28.1.1860

[28] Guan Y, Shortridge KF, Krauss S, Chin PS, Dyrting KC, et al (2000) H9N2 influenza viruses possessing H5N1-like internal genomes continue to circulate in poultry in southeastern China. J Virol 74: 9372–9380. 11000205 10.1128/jvi.74.20.9372-9380.2000 PMC112365

[29] Webster RG, Bean WJ, Gorman OT, Chambers TM, Kawaoka Y (1992) Evolution and ecology of influenza A viruses. Microbiol Rev 56: 152–79. 1579108 10.1128/mr.56.1.152-179.1992 PMC372859

[30] Sun Y, Pu J, Jiang Z, Guan T, Xia Y, et al (2010) Genotypic evolution and antigenic drift of H9N2 influenza viruses in China from 1994 to 2008. Vet Microbiol 146: 215–225. 20685047 10.1016/j.vetmic.2010.05.010

[31] Guan Y, Shortridge KF, Krauss S, Webster RG (1999) Molecular characterization of H9N2 influenza viruses: were they the donors of the “internal” genes of H5N1 viruses in Hong Kong? Proc Natl Acad Sci U S A 96: 9363–9367. 10430948 10.1073/pnas.96.16.9363 PMC17788

[32] Xu KM, Li KS, Smith GJ, Li JW, Tai H, et al (2007) Evolution and molecular epidemiology of H9N2 influenza A viruses from quail in southern China, 2000 to 2005. J Virol 81: 2635–2645. 17192315 10.1128/JVI.02316-06 PMC1865985

[33] Gaidet N, Cattoli G, Hammoumi S, Newman SH, Hagemeijer W, et al (2008) Evidence of infection by H5N2 highly pathogenic avian influenza viruses in healthy wild waterfowl. PLoS Pathog 4: e1000127. 18704172 10.1371/journal.ppat.1000127 PMC2503949

[34] Londt BZ, Banks J, Alexander DJ (2007) Highly pathogenic avian influenza viruses with low virulence for chickens in in vivo tests. Avian Pathol 36: 347–350. 17899457 10.1080/03079450701589134

[35] Kim HR, Park CK, Oem JK, Bae YC, Choi JG, et al (2010) Characterization of H5N2 influenza viruses isolated in South Korea and their influence on the emergence of a novel H9N2 influenza virus. J Gen Virol 91: 1978–1983. 20392898 10.1099/vir.0.021238-0

[36] Lee MS, Chang PC, Shien JH, Cheng MC, Shieh HK (2001) Identification and subtyping of avian influenza viruses by reverse transcription-PCR. J Virol Methods 97: 13–22. 11483213 10.1016/s0166-0934(01)00301-9

[37] Qiu BF, Liu WJ, Peng DX, Hu SL, Tang YH, et al (2009) A reverse transcription-PCR for subtyping of the neuraminidase of avian influenza viruses. J Virol Methods 155: 193–198. 18984006 10.1016/j.jviromet.2008.10.001

[38] Hoffmann E, Stech J, Guan Y, Webster RG, Perez DR (2001) Universal primer set for the full-length amplification of all influenza A viruses. Arch Virol 146: 2275–2289. 11811679 10.1007/s007050170002

[39] Reed LJ, Muench H (1938) A simple method of estimating fifty percent endpoints. AmJEpidemiol 27: 493–497.

[40] Lu X, Tumpey TM, Morken T, Zaki SR, Cox NJ, et al (1999) A mouse model for the evaluation of pathogenesis and immunity to influenza A (H5N1) viruses isolated from humans. J Virol 73: 5903–5911. 10364342 10.1128/jvi.73.7.5903-5911.1999 PMC112651

[41] Perkins LE, Swayne DE (2002) Pathogenicity of a Hong Kong-origin H5N1 highly pathogenic avian influenza virus for emus, geese, ducks, and pigeons. Avian Dis 46: 53–63. 11924603 10.1637/0005-2086(2002)046[0053:POAHKO]2.0.CO;2

